# Supplementary material for: Cloud BioLinux: pre-configured and on-demand bioinformatics computing for the genomics community
Source: BMC Bioinformatics. 2012 Mar 19;13:42. doi: 10.1186/1471-2105-13-42 (PMC3372431; doi:10.1186/1471-2105-13-42)
Supplement: Additional file 1 — Supplementary 1 Cloud BioLinux software documentation in the form of a mini, self-contained website. Users need to download and uncompress the .zip file, and open through a web browser the "index.html" file available on the main directory. (ZIP 1823 kb). [file 1471-2105-13-42-S1.ZIP › Cloud-BioLinux-Package-Documentation/docs/garlic.html]

Bio-Linux Software Documentation Pages

Back to search form

## garlic

|  |  |
| --- | --- |
| Name | garlic |
| Description | Garlic is written for the investigation of membrane proteins. It may be used to visualize other proteins, as well as some geometric objects. This version of garlic recognizes PDB format version 2.1. Garlic may also be used to analyze protein sequences. The garlic package is prepared by Debian-Med and can be installed in Bio-Linux by typing: `sudo apt-get install garlic` For further information about Garlic please see the remote documentation links |
| Homepage | http://www.zucic.org/garlic/ |
| Remote Documentation | http://www.zucic.org/garlic/docs.html |
